# Adult-onset chronic nonbacterial osteitis mimicking refractory axial psoriatic arthritis: successful treatment with combined IL-1/IL-17 inhibition: a case report and brief literature review

**DOI:** 10.1016/j.ero.2026.100262

**Published:** 2026-10-23

**Authors:** Alican Karakoc, Andreas Wirsching, Laura Bucci, Giulia Corte, Armin Atzinger, Georg Schett, Filippo Fagni

**Affiliations:** 1 Department of Internal Medicine 3, Friedrich-Alexander University Erlangen-Nürnberg and Universitätsklinikum Erlangen, Erlangen, Germany; 2 Deutsches Zentrum Immuntherapie, Friedrich-Alexander University Erlangen-Nürnberg and Universitätsklinikum Erlangen, Erlangen, Germany; 3 Department of Nuclear Medicine, Friedrich-Alexander University Erlangen-Nürnberg and Universitätsklinikum Erlangen, Erlangen, Germany

## Abstract

We report a 46-year-old man with palmoplantar pustular psoriasis and severe axial inflammation who was initially diagnosed with refractory axial psoriatic arthritis. Despite sequential treatment with multiple biologic and targeted therapies, disease activity persisted. Reassessment based on anterior chest wall involvement, characteristic positron emission tomography/computed tomography findings, human leukocyte antigen B27 negativity, and exclusion of infectious, malignant, and monogenic autoinflammatory disorders supported a diagnosis of adult-onset chronic nonbacterial osteitis (CNO). The patient showed a marked clinical, laboratory, and imaging response to interleukin (IL)-1 blockade, and sustained disease control was achieved with combined IL-1/IL-17 inhibition. This case highlights the diagnostic challenges of adult CNO and suggests a potential role for dual cytokine inhibition in refractory disease. A brief literature review on current treatment options is provided.

## INTRODUCTION

In this case report, we describe an adult-onset chronic nonbacterial osteitis (CNO) case characterised by palmoplantar pustulosis (PPP) and severe axial involvement, refractory to multiple biologic therapies but successfully treated with combined interleukin (IL)-1 and IL-17 blockade.

## CASE REPORT

A 46-year-old man initially presented with palmoplantar pustular skin lesions followed by inflammatory back pain. Cervical and thoracic magnetic resonance imaging (MRI) performed at the referring centre revealed multiple syndesmophytes, active corner lesions, and bone marrow oedema. Rheumatologic markers, including rheumatoid factor, anticitrullinated protein antibodies, and human leukocyte antigen (HLA-B27), were negative. After exclusion of infectious process, the patient was diagnosed with psoriatic arthritis (PsA) with axial involvement. He was treated sequentially with adalimumab, secukinumab (150 mg every 4 weeks), and etanercept without clinical improvement in joint symptoms.

The patient was referred to our centre in April 2019 with similar complaints. Apart from weight loss, systemic evaluation was unremarkable. Physical examination revealed increased dorsal kyphosis and PPP; peripheral arthritis, uveitis, inflammatory bowel disease, scalp involvement, and nail involvement were absent. Laboratory evaluation showed elevated acute phase reactants (C-reactive protein [CRP], 18.1 mg/L; erythrocyte sedimentation rate, 30 mm/h). Sacroiliac MRI demonstrated active sacroiliitis (Figure 1A); thus, secukinumab (300 mg every 4 weeks) was initiated as first-line therapy. After 6 months of secukinumab therapy, the skin lesions had improved, but axial symptoms persisted without significant change. Because of intolerance to multiple nonsteroidal anti-inflammatory drugs (NSAIDs), the patient required tramadol, cannabidiol vaporisation for pain control and 10 mg prednisolone per day to control axial symptoms. Because of the patient’s concomitant osteoporosis, oral bisphosphonate therapy with alendronate 70 mg/wk was initiated.

**Figure 1:**
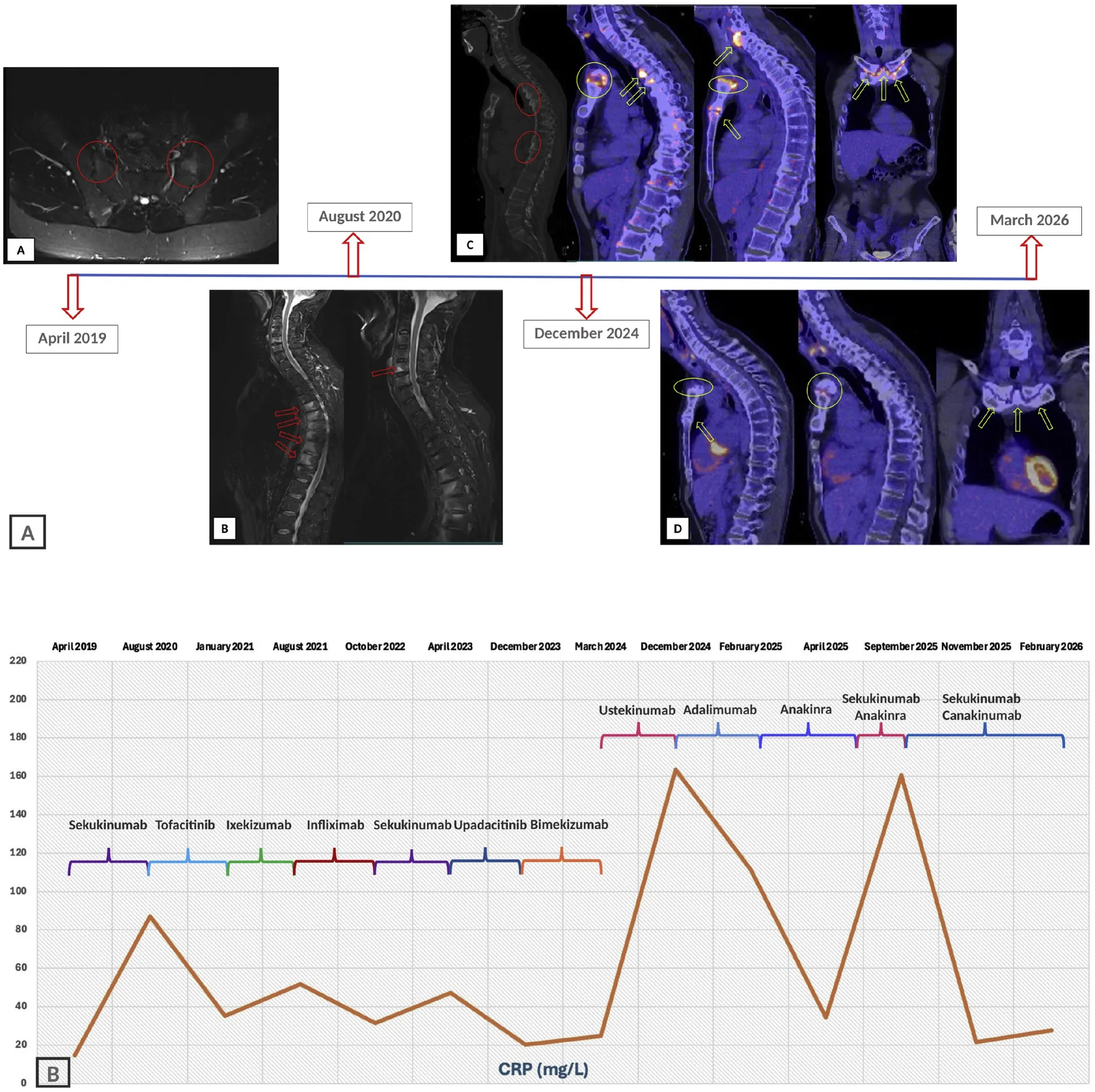
(A) (a) Sacroiliac MRI (STIR) demonstrating signal hyperintensity consistent with bone marrow oedema in the sacroiliac joints (red circles). (b) Cervical and thoracic MRI (STIR) showing signal hyperintensity consistent with extensive bone marrow oedema and corner lesions (red arrows). (c) PET/CT demonstrating intense FDG uptake in the cervical and thoracic vertebrae and in the sternoclavicular and sternomanubrial joints (yellow circles and arrows); CT slices show sclerosis of the thoracic vertebrae (red circles). (d) Follow-up PET/CT during combined IL-1/IL-17 inhibition demonstrating reduced FDG uptake in the spine and the sternoclavicular and sternomanubrial joints (yellow circles and arrows). (B) Longitudinal changes in CRP levels during sequential therapeutic interventions between 2019 and 2026. CRP, C-reactive protein; FDG, fluorodeoxyglucose; IL, interleukin; MRI, magnetic resonance imaging; PET/CT, positron emission tomography/computed tomography; STIR, short tau inversion recovery.

In August 2020, whole-spine MRI revealed extensive bone marrow oedema involving the vertebral corners and vertebral bodies of the cervical and thoracic spine (Fig. 1A) despite therapy with secukinumab and prednisolone. Janus kinase (JAK) inhibition with tofacitinib (10 mg/d) was initiated as second-line, but failed due to primary lack of response. This was followed sequentially by ixekizumab, which controlled skin manifestations but not axial manifestations, and infliximab. Infliximab 300 mg every 6 weeks initially led to skin response and partial improvement of axial symptoms. After a year of infliximab therapy, in October 2022, severe neck and back pain, prolonged morning stiffness, and recurrence of psoriatic lesions despite dosage increase up to 500 mg suggested secondary infliximab failure (CRP, 31.4 mg/L; Bath Ankylosing Spondylitis Disease Activity Index, 7.2; Ankylosing Spondylitis Disease Activity Score with CRP, 4.84). Subsequently, secukinumab therapy was tried again for a short time, followed by upadacitinib, bimekizumab, ustekinumab, and adalimumab. However, axial pain and inflammation on imaging persisted without significant changes under each therapy, and the patient continued to require daily opioid analgesics and prednisolone at a maintenance dose of 10 mg/d. During flares, the prednisolone dose was increased to 30 mg/d and tapered back to the previous dose over the following 2 to 3 weeks.

In December 2024, the patient presented with severe neck and back pain, sternoclavicular and sternomanubrial joint swelling, and weight loss. CRP was 163 mg/L. Extensive screening for infection and malignancy, including blood cultures, echocardiography, immunoelectrophoresis, QuantiFERON testing, and chest CT, was unremarkable. Fluorodeoxyglucose–positron emission tomography/computed tomography (^18^FDG-PET/CT) demonstrated no FDG-avid lesions suggestive of malignancy but showed increased FDG uptake in multiple cervical and thoracic vertebral syndesmophytes/osteophytes and in the sternoclavicular joints (Fig. 1A). These lesions appeared thicker and showed more intense FDG uptake than typical syndesmophytes observed in spondyloarthritis. These findings were consistent with osteitis.

After re-evaluation of the patient, the combination of anterior chest wall involvement, PPP, HLA-B27 negativity, the FDG-PET/CT pattern, and resistance to multiple biologics raised suspicion for CNO. Therefore, genetic testing was requested. Gene panels for autoinflammatory diseases (ADA, APOA1, APOA2, APOC, COPA, ELANE, FGA, GSN, LYZ, MVK, LPIN2, MEFV, NLRP, NOD2, OSMR, OTULIN, PLCG2, PSMB8, PSTPIP1, RELA, STING1, TNFRSF1A, TTR, and UBA1), including mutations involving the IL-1 and IL-36 signalling pathways (IL1RN and IL36RN), all resulted negative.

The diagnosis was revised to CNO, and treatment with anakinra (100 mg/d) and methylprednisolone (30 mg/d) was initiated in January 2025. Intravenous bisphosphonate therapy was recommended as a therapeutic option for CNO; however, treatment was refused because of patient preference for oral bisphosphonate and refusal of intravenous therapies. One week after the initiation of anakinra and glucocorticoid therapy, clinical improvement was observed. Glucocorticoids were gradually tapered and successfully discontinued after one month for the first time without relapse. By month 3, CRP decreased from 111 to 34.5 mg/L, accompanied by marked reductions in disease activity (Bath Ankylosing Spondylitis Disease Activity Index, 8.7→5.8; Ankylosing Spondylitis Disease Activity Score with CRP, 6.08→4.1), allowing discontinuation of tramadol (Fig. 1B).

In September 2025, recurrence of psoriasis and sternoclavicular arthritis led to the addition of secukinumab (300 mg every 4 weeks). Due to injection-site reactions, anakinra was switched to canakinumab (150 mg every 12 weeks). At follow-up in March 2026, the control ^18^FDG PET/CT showed that FDG uptake at the sternoclavicular joints, manubriosternal junction, and spine was markedly reduced (Fig. 1A). The patient remains stable on combination IL-1/IL-17 therapy in near-complete remission. To date, no serious infections were observed under combined cytokine inhibition.

## DISCUSSION

To better define diseases characterised by sterile bone inflammation in adults, the umbrella term ‘chronic nonbacterial osteitis’ has been proposed [1]. CNO represents a disease spectrum affecting primarily the appendicular skeleton in children and the anterior chest wall and axial skeleton in adults. It is frequently associated with skin manifestations, such as palmoplantar pustular pustulosis, psoriasis, acne, and hidradenitis suppurativa, as well as seronegative spondyloarthritis features including sacroiliitis, enthesitis, and dactylitis. Inflammatory bowel disease may occur less frequently, whereas uveitis is generally not expected. Synovitis, Acne, Pustulosis, Hyperostosis, and Osteitis (SAPHO) syndrome is also considered part of the spectrum of sterile inflammatory bone disorders. Although both entities are characterised by sterile osteitis, bone manifestations generally predominate in CNO, whereas SAPHO syndrome is more commonly associated with prominent cutaneous manifestations, particularly PPP and severe acne.

Innate immune dysregulation, particularly involving monocytes, is thought to play a central role in CNO pathogenesis. Monocytes from patients with CNO show increased expression of proinflammatory cytokines (IL-1β, IL-6, and tumour necrosis factor α [TNF-α]) and decreased anti-inflammatory cytokines (IL-10 and IL-19) [2]. The reduced IL-10 and IL-19 expression has been linked to impaired Sp-1 recruitment to their regulatory regions, leading to NLRP3 inflammasome activation, increased IL-1β production, and ultimately contributing to inflammatory bone loss [3]. This chronic inflammatory state promotes osteoclast activation through the receptor activator of nuclear factor-κB and its ligand pathway, resulting in structural bone damage such as sclerosis and ankylosis [4].

Evidence guiding the treatment of adult CNO is largely derived from case reports, case series, and retrospective cohort studies rather than from randomised controlled trials. NSAIDs are recommended as first-line therapy. In patients with an inadequate response, intravenous bisphosphonates or cytokine inhibitors, particularly TNF inhibitors (TNFi), are recommended as second-line treatment, whereas combination therapy with TNFi and intravenous bisphosphonates may be considered in more refractory cases [1,5,6].

A recent German multicentre study evaluated a relatively large cohort of 114 adults with CNO. The cohort was predominantly female (75%), with sternocostoclavicular osteitis (89%) representing the most common skeletal manifestation and PPP (58%) the most frequent extraosseous manifestation. Notably, 70% of patients had overlapping axial spondyloarthritis (axSpA) or PsA. Although NSAIDs were widely used as first-line therapy, only 20% of patients received the recommended dose and duration according to the 2024 international consensus. Conventional disease-modifying antirheumatic drugs were frequently used despite their limited efficacy. TNFi were the most commonly used second-line biologics, whereas intravenous bisphosphonates were used only infrequently [7].

Our patient shared several clinical characteristics with this cohort, including sternocostoclavicular involvement, PPP, and fulfilled the Assessment of SpondyloArthritis international Society classification criteria for axial spondyloarthritis [8] and the Classification Criteria for Psoriatic Arthritis classification criteria for PsA [9]. However, the therapeutic course differed substantially. Despite sequential treatment with multiple biologic agents, the patient's axial symptoms remained refractory. In contrast, initiation of IL-1 blockade resulted in marked clinical improvement, with a dramatic reduction in acute-phase reactants, allowing discontinuation of both glucocorticoids and opioid analgesics. Although IL-1 blockade successfully controlled the patient’s axial symptoms, additional therapy became necessary following the development of PPP and sternocostoclavicular arthritis. IL-17 inhibition was therefore added back in combination with IL-1 blockade to address the persistent cutaneous and peripheral musculoskeletal manifestations. This strategy was supported by the patient's previous favourable cutaneous response to IL-17 blockade and the inadequate efficacy of TNF, JAK, and IL-12/23 inhibitors in controlling both the cutaneous and musculoskeletal manifestations. Although intravenous bisphosphonates are recommended by the international consensus for refractory adult CNO [1], treatment was declined on several occasions due to patient preference, prompting the use of oral bisphosphonates as an alternative.

In our case, the combination of PPP, anterior chest wall and axial involvement, absence of uveitis, HLA-B27 negativity, and exclusion of monogenic autoinflammatory diseases strongly supported the diagnosis of a CNO spectrum disease. The dramatic clinical response to IL-1 blockade highlights the central role of IL-1β, which is consistent with emerging evidence supporting the use of IL-1 inhibitors in paediatric CNO [10]. Notably, IL-1 inhibition has shown some efficacy in SpA [11], although the available evidence has been insufficient to support further clinical development.

Although the role of IL-17 in CNO remains unclear, the frequent coexistence of pustular psoriasis suggests that targeting this pathway may represent an additional therapeutic option in patients with refractory skin disease. Notably, a systematic review reported on 8 cases of SAPHO syndrome successfully treated by IL-17 blockade with secukinumab, particularly for cutaneous manifestations and anterior chest wall involvement [12].

Evidence regarding the use of JAK inhibitors in adult CNO/SAPHO syndrome remains limited to small case series and case reports. Li et al. [13] evaluated the efficacy of tofacitinib for nail involvement in 13 patients with SAPHO syndrome in a single-centre, open-label prospective pilot study. After 12 weeks, significant improvements were observed in nail and skin manifestations, whereas the initial improvement in osteoarticular pain was not sustained. Similarly, Liu et al. [14] evaluated the efficacy of baricitinib in a retrospective case series of 5 patients with SAPHO syndrome and reported improvements in cutaneous manifestations, inflammatory markers, and osteoarticular symptoms. In our patient, both tofacitinib and upadacitinib were attempted; however, neither treatment resulted in improvement in cutaneous manifestations, inflammatory markers, or osteoarticular symptoms.

Although CNO shares several clinical and radiological features with PsA and axSpA, it is considered a distinct autoinflammatory bone disorder with differences in disease course, patterns of musculoskeletal and cutaneous involvement, and therapeutic response. Recognition of these differences is essential because they have important diagnostic and therapeutic implications. The similarities and distinguishing features of these 3 conditions are summarised in Table 1 [1,2,3,7,[15], [16], [17], [18], [19], [20], [21], [22], [23], [24], [25], [26]].

**Table 1:** Comparison of clinical, imaging, and immunological features in axSpA, PsA, and adult CNO

Category	Feature	axSpA	PsA	Adult CNO
Clinical	Age of onset	Young adults	Adults	Adults
	Sex	Male predominance	Equal/slight female	Female predominance
	Axial involvement	+++	++	++
	Peripheral arthritis	+	+++	+
	Enthesitis	+++	+++	+
	Dactylitis	–	+++	–
	Skin involvement	Rare	Psoriasis +++	PPP/acne +
	Anterior chest wall involvement	+	+	+++
	Sacroiliitis	+++	++	+
	Uveitis	++	+	Rare
	IBD association	++	+	Rare
Imaging	Syndesmophytes	Thin/marginal	Bulky/paramarginal	Atypical
	Osteitis	+	+	+++
	Erosions	+	++	++
	New bone formation	+++	+++	++
	^18^FDG PET/CT uptake	Mild-moderate	Mild-moderate	Mild-moderate
Immunology	TNF-α	+++	+++	++
	IL-17	+++	+++	+
	IL-23	++	+++	?
	IL-1β	+	+	+++
	IL-6	+	+	++
	Innate immunity	+	++	+++
	NLRP3 inflammasome	+	+	+++
Treatment	NSAIDs	+++	++	+++
	TNF inhibitors	+++	+++	++
	IL-17 inhibitors	+++	+++	±
	IL-12/23 inhibitors	–	+++	±
	IL-6 inhibitors	–	–	-
	IL-1 blockade	–	–	+
	JAK inhibitors	+++	+++	+
	Bisphosphonates	–	–	++

–, not present/not effective; +, present/effective; +/–, single cases; axSPA, axial spondyloarthritis; CNO, chronic nonbacterial osteitis; FDG, fluorodeoxyglucose; IBD, inflammatory bowel disease; IL, interleukin; JAK, Janus kinase; NLPR3, nod-like receptors; NSAIDs, nonsteroidal anti-inflammatory drugs; PET/CT, positron emission tomography/computed tomography; PPP, palmoplantar psoriasis; PsA, psoriatic arthritis; TNF, tumour necrosis factor.

The comparative ratings (+++, ++, +, +/–) represent the authors’ synthesis of the published literature and expert consensus rather than quantitative prevalence estimates.

Clinical features: axSpA; ref [8], PsA; ref [15], adult CNO; ref [1,7].

Imaging features: axSpA; ref [[16], [17], [18]], PsA; ref [19,20], adult CNO; ref [1,21,22].

Immunological features: axSpA; ref [23] PsA; ref [24], adult CNO; ref [2,3].

Treatment principles: axSpA; ref [25], PsA; ref [26], adult CNO; ref [1,7].

Another notable aspect of this case is the successful use of dual biologic therapy. Although the concomitant use of 2 biologic agents is generally not recommended, it may be considered in carefully selected patients with refractory disease in whom sequential targeted therapies have failed. In the present case, IL-1 blockade effectively controlled the axial inflammatory component, whereas the subsequent addition of IL-17 inhibition achieved control of persistent cutaneous and sternocostoclavicular manifestations, resulting in sustained clinical remission. Consequently, dual cytokine inhibition with IL-1 and IL-17 blockade represented an individualised therapeutic approach that achieved sustained disease control in an otherwise severely refractory patient. To our knowledge, this is the first reported case of CNO successfully treated with dual biologic therapy.

In conclusion, CNO can easily be overlooked in adult patients, particularly in the presence of overlapping rheumatologic features such as psoriasis and sacroiliitis. Anterior chest wall involvement together with pustular psoriasis and axial skeletal inflammation should prompt consideration of CNO in the differential diagnosis. Early recognition and treatment may be crucial for achieving disease control, preserving functional status, and preventing permanent structural damage and chronic pain.

## Funding

This research did not receive any specific grant from funding agencies in the public, commercial, or not-for-profit sectors.

## CRediT authorship contribution statement

**Alican Karakoc:** Writing – original draft, Investigation, Visualization, Writing – review & editing, Data curation, Project administration. **Andreas Wirsching:** Writing – review & editing. **Laura Bucci:** Writing – review & editing. **Giulia Corte:** Writing – review & editing. **Armin Atzinger:** Writing – review & editing, Visualization. **Georg Schett:** Writing – review & editing, Conceptualization, Supervision. **Filippo Fagni:** Writing – review & editing, Conceptualization, Investigation, Supervision, Project administration.

## Competing interests

All authors declare they have no competing interests.
